# A comparative study of recombinant and native frutalin binding to human prostate tissues

**DOI:** 10.1186/1472-6750-9-78

**Published:** 2009-09-09

**Authors:** Carla Oliveira, José A Teixeira, Fernando Schmitt, Lucília Domingues

**Affiliations:** 1 IBB - Institute for Biotechnology and Bioengineering, Centre of Biological Engineering, Universidade do Minho, Campus de Gualtar, 4710-057 Braga, Portugal; 2 IPATIMUP (Institute of Molecular Pathology and Immunology of the University of Porto) and Medical Faculty of Porto University, Rua Dr. Roberto Frias s/n, 4200-465, Porto, Portugal

## Abstract

**Background:**

Numerous studies indicate that cancer cells present an aberrant glycosylation pattern that can be detected by lectin histochemistry. Lectins have shown the ability to recognise these modifications in several carcinomas, namely in the prostate carcinoma, one of the most lethal diseases in man. Thus, the aim of this work was to investigate if the α-D-galactose-binding plant lectin frutalin is able to detect such changes in the referred carcinoma. Frutalin was obtained from different sources namely, its natural source (plant origin) and a recombinant source (*Pichia *expression system). Finally, the results obtained with the two lectins were compared and their potential use as prostate tumour biomarkers was discussed.

**Results:**

The binding of recombinant and native frutalin to specific glycoconjugates expressed in human prostate tissues was assessed by using an immuhistochemical technique. A total of 20 cases of prostate carcinoma and 25 cases of benign prostate hyperplasia were studied. Lectins bound directly to the tissues and anti-frutalin polyclonal antibody was used as the bridge to react with the complex biotinilated anti-rabbit IgG plus streptavidin-conjugated peroxidase. DAB was used as visual indicator to specifically localise the binding of the lectins to the tissues. Both lectins bound to the cells cytoplasm of the prostate carcinoma glands. The binding intensity of native frutalin was stronger in the neoplasic cells than in hyperplasic cells; however no significant statistical correlation could be found (*P *= 0.051). On the other hand, recombinant frutalin bound exclusively to the neoplasic cells and a significant positive statistical correlation was obtained (*P *< 0.00001). However, recombinant frutalin did not recognise all malignant prostate cases and, when positive, the binding to those tissues was heterogeneous.

**Conclusion:**

Native and recombinant frutalin yielded different binding responses in the prostate tissues due to their differences in carbohydrate-binding affinities. Also, this study shows that both lectins may be used as histochemical biomarkers for the prostate cancer. Moreover, the successful use of a recombinant lectin in immunohistochemical studies of prostate cancer was for the first time demonstrated, highlighting the advantages of using recombinant systems in the preparation of pure lectin samples for diagnostic purpose.

## Background

Prostate carcinoma is one of the most common causes of cancer-related deaths in men older than 50 years of age. It is predominantly a disease of older men, with a peak incidence between the ages of 65 and 75 years. Although the cause of prostate cancer is still unknown, clinical and experimental observations suggest that hormonal, genetic, diet and environmental factors, may play a role in its pathogenesis [1-6]. Carcinomas of the prostate are often clinically silent, especially during their initial stages, thus all efforts made towards early diagnosis and therapeutic investigations are important for the treatment of this malignant disease.

Cellular glycoconjugates are localised intracellularly, extracellularly and also at the cell surface, where they can be secretory or structural. Glycoproteins of the cell surface play different roles in cell function including cell differentiation, adhesion, proliferation, morphological changes and functional modulation. Glycosylation plays a major role by determining and stabilising protein folding, modelling of physicochemical properties and determining cell immunogenicity [7]. In the case of the prostate, complex glycans are important for the functional activity of this organ [8]. Glycoconjugates undergo modification associated with the cellular functions perform by them and also under pathological conditions. The glycosylation profile on tumour cells glycoproteins is distinctly different from that on normal cells, due to the blockage of the glycosylation pathways. This phenomenon results in the incomplete elongation of *O*-glycans and premature sialylation, leading to the expression of shorter and modified cancer-associated oligosaccharides such as T, sialyl-T, Tn, sialyl-Tn, LewisX (LeX) and sialyl-LeX, with a drastic impact in the cells normal function [9,10].

Lectins are proteins of non-immune origin that reversibly bind specific carbohydrates and that may also agglutinate cells, precipitate polysaccharides or glycoconjugates [11]. They are widely used in biomedical diagnostic research as histochemical probes to localise and characterise specific carbohydrates residues or oligosaccharides in cells and tissues [12]. Lectin studies conducted in the human prostate, and in its secreted glycoproteins (*e.g. *PSA - prostate-specific antigen), have demonstrated their applicability as biomarkers of specific secretory functions, structural components and developmental alterations of this gland [13-16]. Additionally, lectins histochemical studies were performed to detect changes in glycoconjugates associated with pre-neoplasic and neoplasic transformation of the prostate [8,17]. Frutalin is a tetrameric jacalin-related lectin expressed in the breadfruit plant seeds (*Artocarpus incisa*) that specifically recognises α-D-galactose residues [18]. Frutalin may be successfully used in immunobiological research, on the recognition of cancer-associated oligosaccharides, similarly to other galactose-binding lectins [19]. However, the use of lectins isolated from their natural sources presents several disadvantages, namely the heterogeneity of the sample due to the presence of lectin isoforms, which may lead to results variability. One way to overcome these limitations is a recombinant expression and production, which might improve and facilitate lectin availability and offer a higher level of purity and control of the lectin final properties.

In this work, the expression of frutalin specific glycoconjugates in human tissues of prostate carcinoma and benign prostate hyperplasia (BPH) was studied by immunohistochemistry. The binding pattern obtained with the breadfruit frutalin and with the recombinant frutalin produced by *Pichia pastoris *in the prostate tissues was compared and their potential use as tumour biomarkers was evaluated. For the first time a lectin from a recombinant source was used in immunohistochemical studies of human prostate tissues.

## Methods

### Tumour tissue blocks origin

Formalin-fixed, paraffin-embedded blocks of 20 cases of prostate carcinoma and 25 cases of BPH were analysed. All the cases were retrieved from the files of the Institute of Molecular Pathology and Immunology of the University of Porto (IPATIMUP), Portugal, during the period of 2004 to 2007. The mean age of the patients with prostate carcinoma was 64 years and of the patients with BPH was 68 years. The twenty carcinoma cases were graded according the Gleason scoring system [20]: 18 cases were Gleason score 7 (3+4), one case was Gleason score 6 (3+3) and other Gleason score 8 (4+4).

### Lectins production/purification and antibodies

Native frutalin was purified from *A. incisa *seeds as previously described [18]. The production and purification of recombinant frutalin in the *P. pastoris *expression system has previously been reported [21]. Polyclonal antibody against native and recombinant frutalin was produced by inoculating New Zealand rabbits with native frutalin. The specificity of this antibody to both lectins was previously confirmed by Western blot analysis [21]. The antibody to rabbit IgG was "Biotinylated Goat Anti-Polyvalent", purchased from LabVision Corporation (Fremont, USA).

### Immunohistochemistry

The localisation of frutalin specific glycoconjugates in the prostate tissues was carried out by using the streptavin-biotin-peroxidase complex immunohistochemical technique. Slides were deparaffinised in xylol and rehydrated in a graded ethanol series. The antigen retrieval was done by incubating slides in boiling 10 mM citrate buffer (pH 6.0) for 30 min, using a microwave at 900 Watts, followed by cooling at room temperature for another 20 min. After washes in PBS buffer (8 g/l NaCl, 0.2 g/l KCl, 2.68 g/l Na_2_HPO_4_·7H_2_O and 0.24 g/l KH_2_PO_4_, pH 7.4 adjusted with HCl), endogeneous peroxidase activity was inactivated by incubating the slides for 10 min in 3% (v/v) H_2_O_2_/methanol. Slides were then incubated with a blocking serum (LabVision Corporation, Fremont, USA) for 10 min, for blockage of non-specific protein binding, and then incubated for 1 h at 37°C with native frutalin (1 μg/ml) or recombinant frutalin (50 μg/ml). After washes in PBS, the slides were incubated overnight at 4°C with polyclonal anti-frutalin antibody (0.5 μg/ml). Afterwards, the slides were washed in PBS and incubated for 10 min at room temperature with biotinylated secondary antibody, following by incubation with streptavidin-conjugated peroxidase solution for another 10 min (LabVision Corporation, Fremont, USA). Diaminobenzidine (DAB) was used as the chromogen and finally, the tissues were counterstained with hematoxylin and covered with a mounting solution (Entellan). In each run a positive control (the binding of native frutalin to a prostate carcinoma tissue) and a negative control (where frutalin was replaced with PBS during the lectin incubation period) were also included. All PBS washes were conducted at room temperature and each wash was done two times for 5 min.

The binding of the lectins to the prostate tissues was revealed by a brown colour, as a result of the DAB reaction with the streptavidin-conjugated peroxidase solution. The staining of the tissues was assessed by one pathologist. The frutalin binding was evaluated by the intensity of its cytoplasmatic staining, being graded as negative (0), low (+), moderate (++) or strong (+++), according to the literature [22].

### Statistical analysis

Results were statistically analysed using the program SPSS for windows (version 16.0). The grades of lectin binding intensity were converted numerically as negative = 0, low = 1, moderate = 2 and strong = 3. Mann-Whitney U analysis was used to compare the global binding intensity obtained for each lectin in the two studied histological groups. Results were only considered statistically significant if *P *< 0.05.

The present study was conducted under the national regulative law for the usage of biological specimens from tumour banks, where the samples are exclusively available for research purposes in the case of retrospective studies.

## Results

The results of the immunohistochemical analysis obtained for the binding of native and recombinant frutalin to the human prostate tissues are summarised in Table 1. Native frutalin bound to all the studied cases of prostate carcinoma (100%; 20/20), as well as to all the studied cases of benign prostate hyperplasia (BPH) (100%; 25/25). On the other hand, recombinant frutalin bound to a part of the studied cases of prostate carcinoma (70%; 14/20) and did not bind to any of the studied cases of BPH (0%; 0/25). As the anti-frutalin antibody also bound to the prostate tissues, its concentration was decreased until no binding was observed. Additionally, negative controls were done (where frutalin was replaced with PBS during the lectin incubation period) to make sure that the binding responses obtained corresponded in fact to frutalin and not to the anti-frutalin antibody.

**Table 1:** Immunohistochemistry results for the binding of native and recombinant frutalin to human prostate tissues

**Histological diagnosis**	**Native frutalin**		**Recombinant frutalin**	
	***n *positive**	**%**	***n *positive**	**%**
Benign prostate hyperplasia	25/25	100	0/25	0

Prostate carcinoma	20/20	100	14/20	70

**Figure 1:**
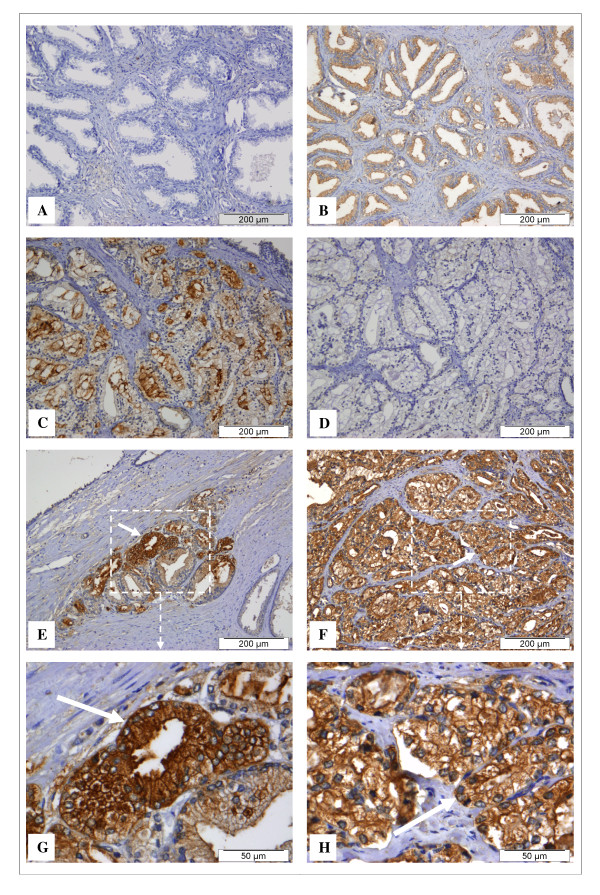
**Detection of frutalin specific glycoconjugates expressed in sections of human prostate tissues by immunohistochemistry**. Samples of formalin-fixed, paraffin-embedded tissues from benign prostate hyperplasia (A, B) and prostate carcinoma (C to H) were immunostained with recombinant frutalin (A, C, E, G) and native frutalin (B, F, H). A negative control proves that the binding responses observed in the prostate carcinoma tissues corresponded to the lectins (D). No detectable recombinant frutalin binding was observed in hyperplasic cells (A), while weak staining was detected for native frutalin (B). Both lectins bound to cytoplasm cells of carcinoma glands (C, E, F, G and H, shown by the white arrows). The binding of recombinant frutalin to the carcinoma tissues was heterogeneous, with closed glands having strong and weak staining (C). The native frutalin staining was strong and homogeneous in carcinoma glands (F and H). Recombinant frutalin was able to specifically recognise carcinoma cells in middle of a benign lesion (E and G).

BPH is a benign lesion of the prostate and it is characterised by the proliferation of both epithelial and stromal elements, with resultant enlargement of the gland (*e.g. *Figure 1A). The prostate carcinomas are composed of small glands that infiltrate the adjacent stroma in an irregular, haphazard fashion (*e.g. *Figure 1D). In contrast to normal and hyperplasic prostate glands, the glands in carcinomas are not encircled by collagen or stromal cells but rather lie "back to back" and appear to dissect sharply though the native stroma. The neoplasic glands are lined by a single layer of cuboidal cells with conspicuous nucleoli and the basal cell layer seen in normal or hyperplasic glands is absent. Native frutalin bound to the cells cytoplasm of both carcinoma (Figure 1F and 1H) and benign prostate hyperplasic glands (Figure 1B). The native frutalin binding pattern was found to be homogeneous in all situations. Frutalin specific sugars were also often detected in the stroma of some malignant prostate tissues (data not shown). As mentioned above, recombinant frutalin did not bind to the benign prostate hyperplasic tissues (Figure 1A). As native frutalin, recombinant frutalin bound to the cells cytoplasm of the carcinoma glands (Figure 1C, E and 1G). The recombinant frutalin binding pattern was heterogeneous, with closed glands having strong and weak staining (Figure 1C), as well as positive and negative areas throughout the tissues.

The statistical analysis of the intensity score of the native and recombinant frutalin binding to the prostate tissues is shown in Table 2. The majority of the malignant cases had a strong native frutalin binding (60%; 12/20), while the benign cases presented mostly a moderate binding for this lectin (52%; 13/25). In spite of decreased intensity of the native frutalin binding in hyperplasic cells, compared to carcinoma cells, no significant correlation was found (*P *> 0.05). Recombinant frutalin binding to malignant cases was mainly low or moderate (65%; 13/20) and did not occur in the benign cases (0%; 0/25). A significant positive association was found between the intensity of the recombinant frutalin binding and the tissues histology (*P *< 0.00001). Since the majority of cases were Gleason 7 and they are distributed along all different intensities of reactions of native and recombinant frutalin staining, we do not find any association between Gleason scoring system and intensity of staining for both lectins.

**Table 2:** Statistical analysis of the binding intensity of native and recombinant frutalin in human prostate tissues

**Frutalin/source**	**Histological diagnosis**	Number of cases for each binding intensity*				**Mann-Whitney U analysis**	
		**0**	**+**	**++**	**+++**	**Mean Rank**	***P***
Native (*A. incisa*)	Benign prostate hyperplasia	0 (0%)	4 (16%)	13 (52%)	8 (32%)	19.90	0.051^I^
	Prostate carcinoma	0 (0%)	1 (5%)	7 (35%)	12 (60%)	26.88	

Recombinant (*P. pastoris*)	Benign prostate hyperplasia	25 (100%)	0 (0%)	0 (0%)	0 (0%)	16.00	0.000^S^
	Prostate carcinoma	6 (30%)	9 (45%)	4 (20%)	1 (5%)	31.75	

*The binding intensity was converted numerically as: negative (0) = 0, low (+) = 1, moderate (++) = 2 and strong (+++) = 3; ^I ^Insignificant statistical result; ^S ^Significant statistical result.

## Discussion

The major sugar residues present in the oligosaccharides of the cell surfaces glycoconjugates are: mannose, N-acetylglucosamine (GlcNac), N-acetylgalactosamine (GalNac), galactose (Gal), fucose (Fuc), and derivatives of sialic acid. The distinct property of lectins to bind specific carbohydrates makes them capable of recognising certain cell surface glycoproteins and detecting alterations on that, by using histochemical techniques. Various lectins have been used as biomarkers of human tumours, on the differentiation of dysplasic/neoplasic cells from normal/hyperplasic cells, as well as on the screening and prognosis of different types of carcinomas.

Several studies have shown that cell transformation into malignancy is closely related to an abnormal expression of carbohydrates on cell surfaces. A lectin immunohistochemical study suggested a strong expression of N-acetylgalactosamine residues in BPH and an increased expression of galactose, fucose and sialic acid residues in prostate carcinoma [8]. Thus, it would be expected that the galactose-binding lectin frutalin would recognize these modifications. Nevertheless, significant differences were found between the binding pattern of native and recombinant frutalin in the prostate tissues. Native frutalin bound to both hyperplasic and carcinoma tissues, while recombinant frutalin only bound to carcinoma tissues. However, the binding of recombinant frutalin to the neoplasic tissues was heterogeneous and did not occur in all studied cases. On the other hand, the binding of native frutalin was homogenous in all cases and very intense in the malignant ones. We have recently reported that native and recombinant frutalin presented a difference of at least 120-fold in carbohydrate-binding affinity [21]. The two lectins showed similar sugar-binding specificity but recombinant frutalin demonstrated less affinity. Thus, it can be concluded that it is the distinct carbohydrate-binding affinity presented by these two lectins that would lead to the different binding responses obtained. This is explained by the molecular differences found between native and recombinant frutalin [21]. Both are tetramer glycoproteins, but native frutalin is made of cleaved α and β chains, while in recombinant these two chains are connected by a linker tetrapeptide, because the cleavage of this linker did not occur in the *P. pastoris *expression system. It has already been reported that the linker cleavage may be needed to improve sugar-binding affinity of galactose-binding jacalin-related lectins [21,23]. Moreover, the *Pichia *glycosylation pattern possibly altered the biological activity of recombinant frutalin as it inhibited the ability to agglutinate rabbit erythrocytes. In this study, recombinant frutalin has unequivocally distinguished between benign and malignant cells, binding exclusively to the neoplasic cells, in contrast to native frutalin. Thus, it can be concluded that the putative diagnostic value of recombinant frutalin may be higher than that for the native lectin. Native frutalin recognised all different histological prostate tissues possibly due to its higher carbohydrate-binding affinity and broad carbohydrate-binding range. In fact, native frutalin is a poly-specific lectin, as showed by sugar-binding fluorescence studies [21]. Native frutalin strongly reacted with the T-antigen disaccharide (Galβ1, 3GalNac) (binding constant in the 10^4 ^M^-1 ^range) while no binding was observed with the recombinant frutalin. Furthermore, the affinity of recombinant frutalin to the monosaccharide Me-α-galactose is in the same magnitude order of the native frutalin affinity to other sugars that recombinant lectin did not recognise, as is the case of D-glucose (binding constants in the 10^2 ^M^-1 ^range). Therefore, the binding of frutalin to specific cancer-associated oligosaccharides could have been masked in prostate carcinoma tissues by the presence of other frutalin binding sugars. Nevertheless, the native frutalin binding responses suggest an increased expression of α-D-galactose residues on the neoplasic cells surface, as its binding was generally more intense in neoplasic cells than in hyperplasic cells, which corroborates previous studies [8].

Several lectins have been used in histochemical studies of the human prostate, to detect glycoconjugate changes related with the malignant transformation of this gland. The most studied lectins were the plant lectins soybean agglutinin (SBA - *Glycine max *agglutinin, specificity: α,βGal, α,βGalNAc) [8,24-28], the gorse (furze) agglutinin (UEA-I - *Ulex europaeus *agglutinin, specificity: α-L-Fuc, GlcNAcβ1, 4GlcNAc) [8,24,26-30], and the peanut agglutinin (PNA - *Arachis hypogaea *agglutinin, specificity: Galβ1,3GalNAc) [8,17,24,26-28,31], which present different carbohydrate-binding specificities. Lectins from other organisms were also studied, such as the animal lectin HPA (garden snail - *Helix pomatia *agglutinin, specificity: αGalNAc, αGlcNAc, αGal) [8] and the fungal lectin AAL (mushroom - *Aleuria aurantia *lectin, specificity: α-L-Fuc) [8,32]. Namely, several studies have demonstrated that the expression of HPA-binding glycoproteins on various human cancers, such as prostate carcinoma, is a marker of metastatic potential and poor prognosis [32].

The plant lectins SBA, UEA-I and PNA, as recombinant and native frutalin, were able to differentiate cells from distinct prostate histological tissues. Namely, SBA and UEA-I showed to be useful indicators of the prostate malignancy, as they distinguished between benign and malignant prostate cells, binding exclusively to dysplasic/carcinoma cells [24,27,28], or binding stronger to these cells, comparing to normal/hyperplasic cells binding [8,26,30]. However, in some studies SBA could not distinguish the referred cells [25,26]. In addition, the PNA lectin bound stronger to neoplasic cells than to normal and hyperplasic cells [8,17,26,27]. Nevertheless, negative binding in some malignant cases was also reported for these lectins, namely for the T-antigen specific PNA lectin (16% of total cases) [31].

It should be noted that all these lectins were obtained from their natural sources and therefore their availability is dependent from a time-consuming process and the final properties can vary as a result of different "batch to batch" isolations. This might have contributed to the variable and contradictory results above described. Frutalin, in particularly, is a heterogeneous mixture of several slightly different amino-acid sequences (isoforms), having or not different glycosylation places and extensions, while recombinant frutalin has a defined amino-acid sequence and therefore its properties are more effective. Frutalin isoforms may have different sugar-binding specificities/affinities and, consequently, variable results can be obtained. Furthermore, recombinant frutalin can be easily produced and purified in the *P. pastoris *heterologous expression system, which is an excellent way for frutalin large-scale production, considering its diagnostic application.

## Conclusion

In this work, the binding of the frutalin lectin, obtained from its natural source and from a recombinant system, to prostate tissues was studied by immunohistochemistry. The results demonstrated that there are alterations in the carbohydrates of cellular glycoconjugates of benign and malignant human prostate tissues, detected by recombinant and native frutalin, namely enhancement of galactose residues in malignancy. The carbohydrate-binding properties of each lectin were responsible for the different binding patterns observed. Native frutalin bound stronger to neoplasic cells than to hyperplasic cells, while recombinant frutalin specifically recognised malignant cells. This study shows that both lectins have a considerable potential value in differentiating neoplasic changes from benign lesions of the human prostate. Namely, recombinant frutalin may be a useful tool to elucidate cases of difficult diagnosis. Moreover, the use of the *P. pastoris *recombinant system for frutalin production is more advantageous than the use of natural sources as the production and purification methodologies are easier and pure homogeneous frutalin samples are obtained.

## Authors' contributions

CO carried out the experimental work and drafted the manuscript. JT participated in the development of the concept. FS participated in the coordination of the study and in the evaluation of the results. LD conceived the study and helped drafting the manuscript. All authors read and approved the final manuscript.
